# A Uniform Linear Multi-Coil Array-Based Borehole Transient Electromagnetic System for Non-Destructive Evaluations of Downhole Casings

**DOI:** 10.3390/s18082707

**Published:** 2018-08-17

**Authors:** Bo Dang, Ling Yang, Changzan Liu, Yahong Zheng, Hui Li, Ruirong Dang, Baoquan Sun

**Affiliations:** 1 Key Laboratory of Education Ministry for Photoelectric Logging and Detecting of Oil and Gas, Xi’an Shiyou University, Xi’an 710065, China; lingyang2915@163.com (L.Y.); hayashy@163.com (Y.Z.); lihuiLeehh@163.com (H.L.); dangrr@xsyu.edu.cn (R.D.); 2 School of Marine Science and Technology, Northwestern Polytechnical University, Xi’an 710072, China; liuchangzan@mail.nwpu.edu.cn; 3 Petroleum Engineering Technology Research Institute, Shengli Oilfield Company, SINOPEC, Dongying 257000, China; bqs_sinopec@126.com

**Keywords:** borehole, transient electromagnetic techniques, non-destructive evaluation, multi-coil array

## Abstract

Borehole transient electromagnetic (TEM) techniques have been proven to be efficient for nondestructive evaluations (NDEs) of metal casings using eddy-current properties. However, physical limitations and bad borehole conditions restrict the use of eddy-current sensors, which makes downhole casing inspections very different from those of conventional NDE systems. In this paper, we present a uniform linear multi-coil array-based borehole TEM system for NDEs of downhole casings. On the basis of the borehole TEM signal model, a numerical multi-coil array approach using the Gauss–Legendre quadrature is derived. The TEM response can be divided into two independent parts related to the transmitting-receiving distance (TRD) and the observation time and casing thickness. Using this property, the signal received by the multi-coil array is weighted to cancel the influence of the TRDs of the different array elements to obtain the optimal response according to the linearly constrained minimum variance criterion, which can be shown to be identical to that of achieving the maximum signal-to-noise ratio. The effectiveness of the proposed method was verified by applying the uniform linear multi-coil array to a borehole TEM system for NDEs of oil-well casings. Field experiments were conducted, and the results demonstrate the effectiveness of the proposed method.

## 1. Introduction

Transient electromagnetic (TEM) techniques have gained much attention over the past several decades owing to their use in a wide range of applications in geophysical prospecting, such as mineral and petroleum exploration [1], geotechnical and environmental investigations [2], and fundamental studies of stress and petrophysics [3]. In the field of borehole detection, TEM systems permit the rapid and accurate acquisition of broad-frequency-range data concerning the electrical and geometrical parameters of each borehole cylindrical layer due to their significant feature of the accessibility to targets [4,5]. This technique, which is also known as transient (pulsed) eddy-current testing [6,7,8], enables highly effective nondestructive evaluations (NDEs) of downhole casings [9,10]. However, unlike surface measurements that use large loop coils [1] or high performance magnetometers [11,12], the structure of borehole TEM sensors is strongly restricted by the physical limitations of the downhole conditions, including limited sensor sizes, low sampling performance due to high temperatures, and cumbrous metal tool housings against the high pressure, thereby, reducing the signal strength, as well as signal-to-noise ratio (SNR) of the borehole TEM measurements, and making NDEs of downhole casings very different.

Much research has previously been undertaken with respect to the design and use of eddy-current sensors to improve the NDE performance for downhole casings. Considering the limited space and the high temperatures, which result in a weak TEM response and low SNR, multi-turn coil approaches have been proposed to enhance the signal strength and to improve the inspection performance. In Ref. [13], the eddy-current NDE of metal pipes with an arbitrary position of the coil sensors was theoretically and experimentally investigated. To inspect different crack shapes, a combination of longitudinal and transverse sensors composed of coaxial multi-turn transmitting and receiving coils wound around a magnetic or air core was employed [14,15], and it is shown in [15] that the longitudinal sensor performs better than the transverse sensor for detection of large area. In Ref. [16], multi-pipe strings, which are required to protect the oil and gas wells from by-products for safety considerations, but which make NDEs more difficult in terms of data interpretation [15,17], were inspected using the eddy-current diffusion properties of a TEM system with a multi-turn coil-based auxiliary sensor in longitudinal direction. The above methods have been proven to be effective for improving the NDE performance in downhole casings. However, restricted by the small radial dimensions of the wellbore, the large number of turns of longitudinal multi-turn coil sensors will also increase the length of sensor, as well as the transmitting–receiving distance (TRD), which will lead to serious distortions in the receiving signal model, and strongly influence the accuracy of NDEs. 

At present, there have been minimal previous attempts to eliminate the influence of TRD on downhole measurements, which is difficult to suppress using the traditional multi-turn coil sensor with single-receiver [13,14,15,16,17], since the TRD is coupled with the electrical and geometrical parameters in only one output. On the basis of Doll’s theory in induction logging system [18], the TRD was replaced by two distances from the transmitting and receiving coils to “an elementary ring”. Using this approximate theory, the multi-coil array sensors were successfully used for magnetic field focusing [19] to improve borehole detection performance [20,21]. This method is valid in an open-hole with sufficiently resistive medium by neglecting the skin effect; however, in a cased-hole with a high-conductivity metal casing, Doll’s theory may not be satisfied. In Ref. [22], a two-coil structure was proposed in the case of motion measurements, where a reference multi-turn receiver was used to cancel the effect of the magnetic background noise in the main channel by employing the correlation of the two receivers with different TRDs. Although the relationship between the two receivers was not investigated thoroughly enough to be extended to a multi-coil array sensor, the use of multiple receivers would offer a new way for solving the problem caused by TRD.

In this paper, we present a uniform linear multi-coil array-based borehole TEM system for NDEs of downhole casings. Using a Gauss–Legendre quadrature-based numerical approach to the borehole TEM signal model, we apply a weight to the signals received by the multi-coil array according to the linearly constrained minimum variance (LCMV) criterion [23]. It is shown that the influence of TRD can be eliminated using LCMV-based array signal processing, as compared to the traditional multi-turn coil sensor with the same total number of receiving coil turns, where the maximum SNR can also be achieved. The effectiveness of the proposed system was verified by applying it to an oil borehole TEM system to inspect an oil-well casing.

The rest of this paper is organized as follows. The borehole TEM signal model based on a linear uniform multi-coil array with a coaxial transmitting coil and multiple receiving coils is presented in Section 2. In Section 3, we present the Gauss–Legendre quadrature-based numerical approach to the borehole TEM signal model. The LCMV-based array signal processing method for the linear uniform multi-coil array of the borehole TEM system and its performance analyses are presented in Section 4. The experimental and simulation results are discussed in Section 5. Finally, we conclude the paper in Section 6.

## 2. Borehole TEM Signal Model

**Figure 1:**
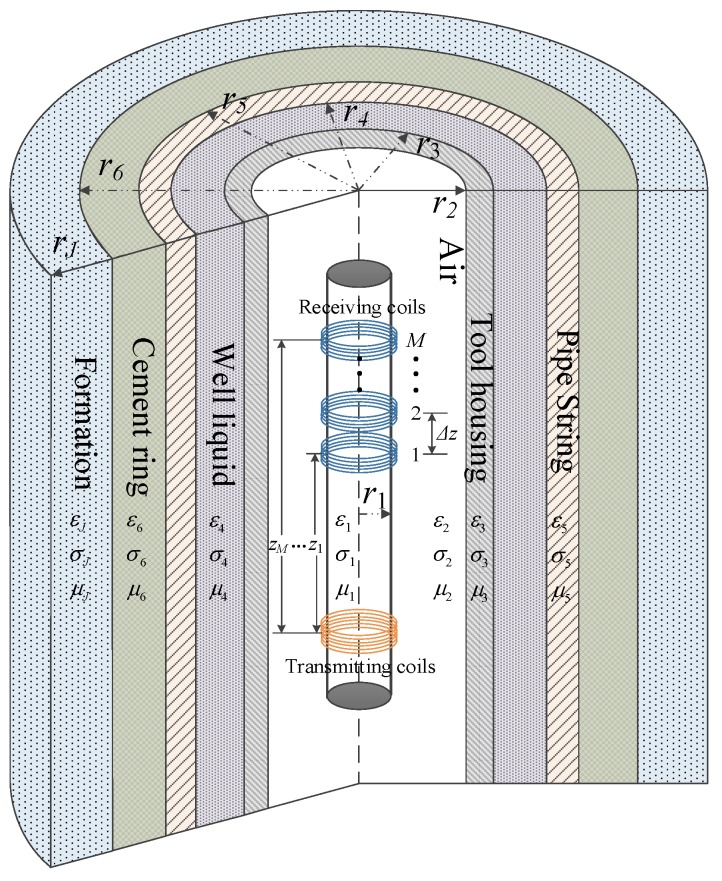
Linear multi-coil array-based borehole transient electromagnetic (TEM) system.

Consider a uniform linear multi-coil array-based borehole TEM system equipped with a single transmitter and *M* receivers with an inter-element spacing of Δ*z* and TRDs ranging from *z*_1_ to *z_M_*, which are comprised of coaxial coils wound around a soft magnetic core in a cylindrically layered medium. The multi-cylindrically layered structures of the borehole TEM system including the electrical and geometrical parameters of the *j*th layer (*μ_j_*, *ε_j_*, *σ_j_*, and *r_j_* with *j* = 1,2,…,*J*) are illustrated in Figure 1. We consider the magnetic core to be the innermost layer. The transmitter and the *M* receivers are located in the second layer, with their number of turns given by *N*_T_ and *N*_R_, respectively, where each receiver has the same number of turns. In this paper, *M* receivers are utilized instead of the traditional single receiver [13,14,15,16,17] with the same total number of receiving coil turns of *MN*_R_. Thereby, with a large value of *M*, *N*_R_, as well as *N*_T_, can be small enough to ensure that the change in the TRD for each single receiver can be ignored and treated approximately as a single value. It is assumed that the diameters of all the coils are sufficiently small so that the source region contains only the second layer, and the induced electromagnetic force (EMF) of the receiving coils is only related to the vertical component of the magnetic field of the first layer (the magnetic core). Moreover, all of the other layers, such as the liquid mud, casing, and formation, are regarded as source-free regions. In addition, to measure the thickness of the metal pipes, we assume that the electrical parameters of all the layers and the inner radius of the metal pipes are fixed.

The response of a TEM system in such a multi-cylindrically layered geometry consists of reflection and transmission components with both standing and outgoing waves. Using the vector potential **A**, the homogeneous and inhomogeneous Helmholtz equations are given by [18]:

(1) $$\nabla^{2}A_{2}+k_{2}^{2}A_{2}=-J_{e},$$

(2) $$\nabla^{2}A_{j}+k_{j}^{2}A_{j}=0,\;\begin{array}{c} j\neq 2 \end{array},$$

where *k_j_*^2^ = *μ_j_ε_j_ω*^2^ − *iμ_j_σ_j_ω* and **J**_e_ denotes the electrical source. With the introduction of the variables *x_j_* and *λ*, which satisfy *x_j_*^2^ = *λ*^2^ − *k_j_*^2^, the vector potential **A** can be calculated by solving the Helmholtz equations. Note that directional measurements cannot be achieved using the proposed borehole TEM system due to the cylindrical symmetry of the model in Figure 1. For the primary field [18], the solution of the inhomogeneous Helmholtz equation in the source region has the form:

(3) $$A_{2}\left(\omega ,z,r\right)=\{\begin{array}{c} \frac{I_{T}r_{0}}{\pi }\int_{0}^{\infty }K_{1}(x_{2}r)I_{1}(x_{2}r_{0})\cos\left(\lambda z\right)d\lambda ,\;r>r_{0}\\ \frac{I_{T}r_{0}}{\pi }\int_{0}^{\infty }K_{1}(x_{2}r_{0})I_{1}(x_{2}r)\cos\left(\lambda z\right)d\lambda ,\;r<r_{0} \end{array},$$

where *I*_T_ denotes the transmitting current, *z* denotes the distance between the excitation (transmitter) and observation (receiver) positions along the borehole axis, *r*_0_ is the radius of the transmitter, and *I*_1_(·) and *K*_1_(·) are modified Bessel functions of the first and second kind of order one, respectively. For the secondary field [18], the solution of the homogeneous Helmholtz equation has the form:

(4) $$A_{j}\left(\omega ,z,r\right)=\frac{I_{T}r_{0}}{\pi }\int_{0}^{\infty }\left[C_{j}I_{1}(x_{j}r_{j})+D_{j}K_{1}(x_{j}r_{j})\right]\cos\left(\lambda z\right)d\lambda ,$$

where *C_j_* and *D_j_* denote the reflection and transmission coefficients, respectively, which are related to the geometrical and electrical parameters of all the layers. Note that *C_J_* and *D*_1_ are zero due to the absence of transmission and reflection in the innermost and outermost layers, respectively. Then, *C_j_* and *D_j_* can be calculated using the boundary conditions of the multilayered cylindrical structures [18]. In the source region, the response contains both primary and secondary fields, while in the source-free region, only the secondary field is involved. Therefore, the vertical component of the magnetic field [18] in the innermost layer with radius *r* (0 < *r* < *r*_1_) can be calculated by combining the solutions of the Helmholtz equations, and can be written as:

(5) $$H_{z1}\left(\omega ,z,d\right)=\frac{N_{T}r_{1}I_{T}}{\pi }\int_{0}^{\infty }x_{1}C_{1}I_{0}\left(x_{1}r\right)\cos(\lambda z)d\lambda ,$$

where *I*_0_(·) denotes a modified Bessel function of the first kind of order zero, and *d* denotes the thickness of the metal casings, and can be expressed by *r*_5_ − *r*_4_ if only one casing is used; all of these geometrical parameters are included in *C*_1_. On the basis of the above models [18], the induced EMF in the frequency domain in the *m*th receiver of multi-coil array with a TRD of *z_m_* can then be expressed as:

(6) $$U_{m}\left(\omega ,z_{m},d_{m}\right)=-i\omega \xi \int_{0}^{r_{1}}\int_{0}^{\infty }2\pi rf(\lambda ,r,\omega ,d_{m})\cos(\lambda z_{m})d\lambda dr,$$

where *ξ = μ*_1_*N*_R_*N*_T_*I*_T_/*π*, *f*(*λ*,*r*,*ω*,*d_m_*) = *x*_1_*C*_1_*I*_0_(*x*_1_*r*), and *d_m_* denotes the thickness of the metal casing with respect to the observation position of the *m*th receiver. Given a ramp signal with a turn-off time of *t*_of_, the induced EMF *U_m_*(*t,z_m_,d_m_*) can be obtained by converting Equation (6) into the time domain. Using the Gaver–Stehfest inverse Laplace transform [24] with *S* stages as an example, we obtain:

(7) $$U_{m}(t,z_{m},d_{m})=\frac{\ln2}{t}\sum_{s=1}^{S}D_{s}\frac{e^{(-s\;\ln2t_{\mathrm{of}})/t}-1}{t_{\mathrm{of}}{\left(s\;\ln2/t\right)}^{2}}\cdot U_{m}(s\;\ln2/it,z_{m},d_{m}),$$

where *iω* = *s*ln2/*t* and *t* and *D_s_* denote the observation time and the integral coefficient of the Gaver–Stehfest inverse Laplace transform, respectively. We can see that the induced EMF of the borehole TEM system is related not only to the observation time and the thickness of the downhole casing, but also to the TRD of the *m*th receiver. In other words, the three variables in Equation (7) are coupled together in the TEM response. Of these three variables, it is the coupling between *t* and *d*, also known as the eddy-current diffusion or the time-domain property, that offers several benefits to the TEM system to achieve better performance [16,25]. Conversely, when a single receiver with a large number of turns or a multi-coil array is used, the TRD will strongly influence the interpretation of the NDE, where the change in the TRD cannot be ignored and must be compensated for to avoid model distortions in the borehole TEM system.

## 3. Numerical Approximation of the Borehole TEM Signal Model

In previous studies, multi-turn coils and multi-coil arrays have been used to improve the inspection performance of borehole TEM systems. However, as shown in Section 2, the effect of the TRD on the TEM response makes the interpretation of NDEs more difficult. In Ref. [22], even though the correlation between two channels with different TRDs is employed for noise cancellation with a registration matrix, this correlation was not thoroughly investigated because the two parts with respect to the TRD and geometrical–electrical parameters are still coupled to each other. In this section, we decouple the TRD and the other two variables in Equation (7) using a Gauss–Legendre quadrature-based [26] numerical approximation for the borehole TEM signal model. Considering the monotonically decreasing characteristic of the integrand in Equation (6) with respect to the modified Bessel functions, the upper limit of the infinite integral can be reduced to an approximately limited value of *λ*_0_ (*λ*_0_ = 6000 in this paper). Then, by adjusting the integration interval of the two integrations, we can rewrite Equation (6) as:

(8) $$U_{m}\left(\omega ,z_{m},d_{m}\right)=-i\omega \xi \chi \int_{-1}^{1}\int_{-1}^{1}\frac{r^{\prime }+1}{2}f(\lambda_{0}\frac{\lambda^{\prime }+1}{2},r_{1}\frac{r^{\prime }+1}{2},\omega ,d_{m})\cos(\lambda_{0}z_{m}\frac{\lambda^{\prime }+1}{2})d\lambda^{\prime }dr^{\prime },$$

where *χ* = *πr*_1_^2^*λ*_0_/2. On the basis of the Gauss–Legendre quadrature equation, the above two integrations can both be expanded as multi-stage Legendre polynomials such that:

(9) $$U_{m}\left(\omega ,z_{m},d_{m}\right)=-i\omega \xi \chi \sum_{q=1}^{Q}A_{q}\frac{B_{q}+1}{2}\sum_{p=1}^{P}A_{p}f(\lambda_{0}\frac{B_{p}+1}{2},r_{1}\frac{B_{q}+1}{2},\omega ,d_{m})\cos(\lambda_{0}z_{m}\frac{B_{p}+1}{2}),$$

where *P* and Q represent the number of stages of the two Legendre polynomials, with their quadrature coefficient and zero-point denoted by *A* and *B*, respectively. Substituting Equation (9) into Equation (7), we have:

(10) $$U_{m}(t,z_{m},d_{m})=-\frac{\xi \chi }{t_{\mathrm{of}}}\sum_{s=1}^{S}\sum_{q=1}^{Q}\sum_{p=1}^{P}g_{s,q,p}\left(t,d_{m}\right)\cdot v_{p}\left(z_{m}\right),$$

where

(11) $$g_{s,q,p}\left(t,d_{m}\right)=D_{s}\frac{e^{(-s\ln2t_{\mathrm{of}})/t}-1}{s}\cdot A_{q}\frac{B_{q}+1}{2}A_{p}f(\lambda_{0}\frac{B_{p}+1}{2},r_{1}\frac{B_{q}+1}{2},s\;\ln2/it,d_{m}),$$

(12) $$v_{p}\left(z_{m}\right)=\cos(\lambda_{0}z_{m}\frac{B_{p}+1}{2}),$$

In matrix form, Equation (10) can be expressed as:

(13) $$U_{m}(t,z_{m},d_{m})=-\frac{\xi \chi }{t_{\mathrm{of}}}\cdot x\left(z_{m}\right)\cdot g^{T}\left(t,d_{m}\right),$$

where

(14) $$x\left(z_{m}\right)={\left[\underset{SQ}{\underset{︸}{v\left(z_{m}\right),\ldots ,v\left(z_{m}\right)}}\right]}_{1\times SQP},$$

(15) $$v\left(z_{m}\right)={\left[v_{1}\left(z_{m}\right),\ldots ,v_{P}\left(z_{m}\right)\right]}_{1\times P},$$

(16) $$g\left(t,d_{m}\right)={\left[g_{1,1,1}\left(t,d_{m}\right),\ldots ,g_{S,Q,P}\left(t,d_{m}\right)\right]}_{1\times SQP}.$$

In Ref. [13], **x**(*z_m_*) and **g**(*t*,*d_m_*) represent the effect of the TRD and the geometrical–electrical parameters of the multi-cylindrical structure, respectively. Using the Gauss–Legendre quadrature-based numerical approach, the two parts can be approximately separated and are independent from each other, which means that the influence of the TRD on the induced EMFs can be eliminated by cancelling **x**(*z_m_*). To formulate a clear expression, we assume that the induced EMFs of each coil are discretely sampled with a sampling length *L* and a sampling interval ∆*t* by a 16-bit analog-to-digital converter; then, the induced EMF of the *m*th receiver with sampling time *t_l_* and TRD *z_m_* can be rewritten as:

(17) $$U_{m,l}(t_{l},z_{m},d_{m})=-\frac{\xi \chi }{t_{\mathrm{of}}}\cdot x\left(z_{m}\right)\cdot g^{T}\left(t_{1}+\left(l-1\right)\Delta t,d_{m}\right).$$

The vector form of the induced EMF of the *m*th receiver can be expressed as:

(18) $$\begin{array}{ll} U_{m,1-L} & =\left[\begin{array}{cccc} U_{m,1}(t_{1},z_{m},d_{m}) & U_{m,2}(t_{2},z_{m},d_{m}) & \ldots & U_{m,L}(t_{L},z_{m},d_{m}) \end{array}\right]\\ & =-\frac{\xi \chi }{t_{\mathrm{of}}}\cdot x\left(z_{m}\right){\left[\begin{array}{cccc} g^{T}\left(t_{1},d_{m}\right) & g^{T}\left(t_{1}+1\cdot \Delta t,d_{m}\right) & \cdots & g^{T}\left(t_{1}+\left(L-1\right)\cdot \Delta t,d_{m}\right) \end{array}\right]}_{1\times L} \end{array},$$

and the matrix form of the received signal of the uniform linear multi-coil array can be expressed by stacking the induced EMF of each receiver, such that:

(19) $$\begin{array}{ll} U_{1-M,1-L} & ={\left[\begin{array}{cccc} U_{1,1-L}^{T}\left(t,z_{1},d_{1}\right) & U_{2,1-L}^{T}\left(t,z_{2},d_{2}\right) & \ldots & U_{M,1-L}^{T}\left(t,z_{M},d_{M}\right) \end{array}\right]}^{T}\\ & =-\frac{\xi \chi }{t_{\mathrm{of}}}\cdot {\left[\begin{array}{cccc} x(z_{1})\cdot g^{T}(t_{1},d_{1}) & x(z_{1})\cdot g^{T}(t_{2},d_{1}) & \cdots & x(z_{1})\cdot g^{T}(t_{L},d_{1})\\ x(z_{2})\cdot g^{T}(t_{1},d_{2}) & x(z_{2})\cdot g^{T}(t_{2},d_{2}) & \cdots & x(z_{2})\cdot g^{T}(t_{L},d_{2})\\ \vdots & \vdots & \ddots & \vdots \\ x(z_{M})\cdot g^{T}(t_{1},d_{M}) & x(z_{M})\cdot g^{T}(t_{2},d_{M}) & \cdots & x(z_{M})\cdot g^{T}(t_{L},d_{M}) \end{array}\right]}_{M\times L} \end{array}$$

In this paper, we assume that the length of the multi-coil array is much smaller than the distance between two neighboring thickness changes, which means that the thicknesses of the metal casing with respect to the observation position of each receiving coil are the same along the borehole axis with *d*_1_ = *d*_2_ =…= *d_M_* = *d*_0_. Then, the received signal of the multi-coil array can be expressed as:

(20) $$U_{1-M,1-L}=-\frac{\xi \chi }{t_{\mathrm{of}}}\cdot \left[\begin{array}{c} x\left(z_{1}\right)\\ x\left(z_{2}\right)\\ \vdots \\ x\left(z_{M}\right) \end{array}\right]\cdot {\left[\begin{array}{c} g\left(t_{1},d_{0}\right)\\ g\left(t_{2},d_{0}\right)\\ \vdots \\ g\left(t_{L},d_{0}\right) \end{array}\right]}^{T}.$$

Considering the system noise, we rewrite the received signals of the *l*th sampling time, as well as the *l*th column of Equation (20) as follows:

(21) $$U_{1-M,l}=-\frac{\xi \chi }{t_{\mathrm{of}}}\cdot X\left(z\right)\cdot g^{T}(t_{l},d_{0})+N,$$

where

(22) $$X\left(z\right)={\left[{\begin{array}{cccc} x{\left(z_{1}\right)}^{T} & x{\left(z_{2}\right)}^{T} & \ldots & x\left(z_{M}\right) \end{array}}^{T}\right]}_{M\times SQP}^{T},$$

(23) $$N={\left[\begin{array}{cccc} n_{1} & n_{2} & \ldots & n_{M} \end{array}\right]}_{M\times 1}^{T}.$$

**N** is assumed to follow a Gaussian distribution, and each element of **N** is independently identically distributed. Obviously, for each sampling time, the difference between each receiver is the TRD. Notably, in Equation (21), the matrix **X**(*z*) plays a similar role as the array manifold of a phased array in radar signal processing, and will contaminate the matrix **g**(*t_l_*,*d*_0_), which includes the thicknesses of the metal casings to be estimated. Moreover, because the array manifold **X**(*z*) is independent of the observation time and the casing thickness, the cancellation of the TRD effect will not impact NDEs of downhole casings, and therefore, offers a new way to process the TEM data collected from the uniform linear multi-coil array with each coil corresponding to different TRDs. In the next section, we use the array signal processing principle to solve this problem on the basis of the proposed numerical approximation.

## 4. LCMV-Based Multi-Coil Array Weighting for a Borehole TEM System

### 4.1. LCMV-Based Multi-Coil Array Weighting Model

**Figure 2:**
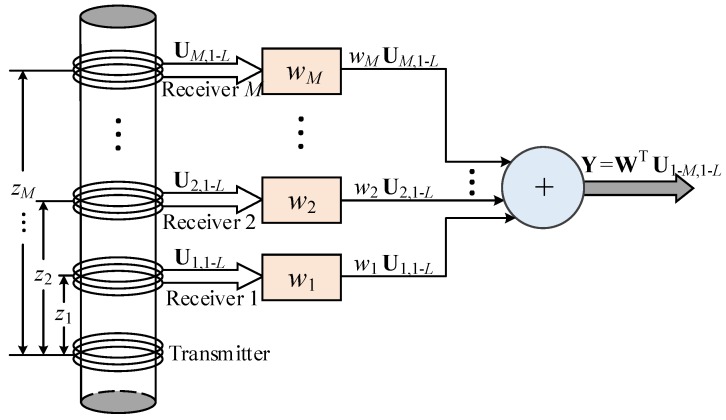
The weighting structure of the linear multi-coil array output.

In Section 3, it was illustrated that **X**(*z*) is only related to the TRD for a uniform linear multi-coil array, and acts similarly to an array manifold in a phased-array radar. Therefore, we can apply a weight to the received signal to eliminate the influence of the TRD by cancelling out the array manifold **X**(*z*). Under this configuration, we employ the principle of the LCMV criterion to weight the array signals in Equation (21), where a receiving weight vector **W**∈*R^M×^^1^* is applied to optimize the array data.

(24) $$y_{l}=-\frac{\xi \chi }{t_{\mathrm{of}}}W^{T}X(z)g^{T}(t_{l},d_{0})+W^{T}N,$$

where *y_l_* is the weighted array output of a sampling time *t_l_*. Note that, when *z* = 0, we have **x**(0) = **F**, and therefore, **Fg**^T^(*t_l_*, *d*_0_) is just the desired part of the received signal of the receiver collocated with the transmitter, where **F**∈*R^1×SQP^* is a row vector with all *SQP* elements equal to 1. Then, according to the LCMV criterion-based adaptive digital beamforming method [23], we let the desired array output of Equation (13) be **Fg**^T^(*t_l_*, *d*_0_). The constraint should be subject to **W**^T^**X**(*z*) = **F**. Moreover, because **X**(*z*) is only related to the TRD, the array weight will not change regardless of the sampling time and casing thickness as long as the multi-coil array structure is fixed. Using this property, the array weighting can be extended by applying the weight to the received signal of the uniform linear multi-coil array for all *L* sampling times such that:

(25) $$Y={\left[\begin{array}{cccc} y_{1} & y_{2} & \ldots & y_{L} \end{array}\right]}_{1\times L}=W^{T}U_{1-M,1-L},$$

where the weighting structure of the proposed method is described in Figure 2.

In this paper, the array weighting can be applied to the output of each receiver by an operation amplifier, where the weight coefficient should be a real value as the cosine but not complex function of **X**(*z*). Then, we can directly optimize the received signal to obtain the optimal array output by minimizing the variance of Equation (24), and the optimal weight can be calculated by using the following optimization problem with the LCMV criterion as

(26) $$\{\begin{array}{c} \min\;W^{T}R_{U}W\\ s.t\;\begin{array}{c} W^{T}X(z)=F \end{array} \end{array},$$

where

(27) $$\begin{array}{ll} R_{U} & =E{\left\{U_{1-M,l}U_{1-M,l}^{T}\right\}}_{M\times M}\\ & =X(z)E\left\{g(t_{l},d_{0})g^{T}(t_{l},d_{0})\right\}X^{T}(z)+R_{N}\\ & =\delta_{s}^{2}X(z)X^{T}(z)+R_{N} \end{array},$$

denotes the auto-correlation matrix of **U**_1-*M*,*l*_ and **R_N_** is the noise correlation matrix (NCM). Moreover, because each channel has the same number of turns of receiving coils, the NCMs of all the channels are also the same.

### 4.2. Approximate Optimal Solution for the LCMV Criterion Problem in Borehole TEM System

Using the Lagrange multiplier method [23], the upper optimization problem can be transformed by introducing the Lagrange operator vector **β**∈C*^1×SQP^*, such that:

(28) $$\Xi =W^{T}R_{U}W+\beta \left(X^{T}(z)W-F^{T}\right),$$

where each of the elements of **β** satisfy 0 *< β_sqp_ <* 1 and the optimal weight is:

(29) $$W=R_{U}^{-1}X(z){\left(X^{T}(z)R_{U}^{-1}X(z)\right)}^{-1}F^{T}.$$

In Equation (29), the rank of **X**^T^(*z*)**R_U_**^−1^**X**(*z*) should be the smaller of *SQP* and *M*. In Section 3, considering the numerical approach that converts the finite integral to a matrix multiplication using the Gauss–Legendre quadrature, larger *P* and *Q* stage numbers will result in smaller approximation errors. Conversely, the manufacture of the multi-coil array will restrict *M*. As a result, *SQP* is usually much larger than *M*, and **X**^T^(z)**R_U_**^−1^**X**(*z*) is not invertible when solving the optimization problem. In this paper, the characteristics of the numerical approximation-based borehole TEM model are used to approximately solve this problem. According to Equation (13), the constraint in Equation (26) can be expanded as:

(30) $$W^{T}X(z)={\left[\begin{array}{c} w_{1}\\ w_{2}\\ \vdots \\ w_{M} \end{array}\right]}^{T}\cdot {\left[\underset{SQ}{\underset{︸}{\begin{array}{cccc} v\left(z_{1}\right) & v\left(z_{1}\right) & \ldots & v\left(z_{1}\right)\\ v\left(z_{2}\right) & v\left(z_{2}\right) & \ldots & v\left(z_{2}\right)\\ \vdots & \vdots & \ddots & \vdots \\ v\left(z_{M}\right) & v\left(z_{M}\right) & \ldots & v\left(z_{M}\right) \end{array}}}\right]}_{M\times SQP}.$$

Equation (30) indicates that the column of **X**(*z*) is repeated with an interval of *P*. Substituting Equation (15) into Equation (30), the constraint of the optimization problem in Equation (30) can be converted to:

(31) $$W^{T}\Lambda \left(z\right)={\left[\begin{array}{c} w_{1}\\ w_{2}\\ \vdots \\ w_{M} \end{array}\right]}^{T}\cdot {\left[\begin{array}{c} v_{1}\left(z_{1}\right),\ldots ,v_{P}\left(z_{1}\right)\\ v_{1}\left(z_{2}\right),\ldots ,v_{P}\left(z_{2}\right)\\ v_{1}\left(z_{M}\right),\ldots ,v_{P}\left(z_{M}\right) \end{array}\right]}_{M\times P}=\Phi ,$$

where **Λ**∈R*^M×P^* and **Φ**∈R^1*×P*^ is a row vector with all *P* elements equal to 1. Now, as long as *M* ≥ *P*, which can be achieved in the fabrication of the multi-coil array sensor, **X**^T^(*z*)**R_U_**^−1^**X**(*z*) can be inverted. Substituting Equation (31) into Equation (29), the optimal weight can then be calculated as:

(32) $$W=R_{U}^{-1}\Lambda \left(z\right){\left(\Lambda^{T}(z)R_{U}^{-1}\Lambda (z)\right)}^{-1}\Phi^{T}.$$

### 4.3. Performance Analyses

On the basis of the LCMV-based multi-coil array for borehole TEM system, we demonstrated that the influence of the TRD can be effectively eliminated, where the received signals of the uniform linear multi-coil array can be weighted to obtain the optimal response that would be received by a receiver with *z* = 0. Now, we show performance analyses of the proposed LCMV-based multi-coil array method. According to Equation (26), the SNR of the received signal of the multi-coil array follows:

(33) $$\mathrm{SNR}=\frac{W^{T}R_{U}W}{W^{T}R_{N}W}.$$

Assuming that the NCM can be decomposed as **R_N_** = **Γ**^T^**Γ**, where **Γ** is invertible, Equation (33) can then be rewritten as:

(34) $$\mathrm{SNR}=\frac{{\left(\Gamma W\right)}^{T}{\left(\Gamma^{-1}\right)}^{T}X(z)X^{T}(z)\Gamma^{-1}(\Gamma W)}{{\left(\Gamma W\right)}^{T}(\Gamma W)}.$$

According to the Schwartz inequality, we have:

(35) $${\left(\Gamma W\right)}^{T}{\left(\Gamma^{-1}\right)}^{T}X(z)X^{T}(z)\Gamma^{-1}(\Gamma W)\leq {\left(\Gamma W\right)}^{T}(\Gamma W)\left(X^{T}(z)\Gamma^{-1}\right){\left(X^{T}(z)\Gamma^{-1}\right)}^{T}.$$

Therefore, the max SNR can only be achieved when (**X**^T^(*z*)**ΓΓ**^−1^)^T^**μ** = **ΓW** and the optimal weight is

(36) $$W_{\mathrm{SNR}}=R_{N}^{-1}X(z)\mu ,$$

where **μ**∈C*^SQP×1^*. Conversely, assuming that the noise and the signal are not correlated, the inversion of **R_U_** can be rewritten as:

(37) $$R_{U}^{-1}=R_{N}^{-1}-\frac{\delta_{s}{}^{2}R_{N}^{-1}X(z)\cdot X^{T}(z)R_{N}^{-1}}{1+\delta_{s}{}^{2}X^{T}(z)R_{N}^{-1}X(z)}.$$

The optimal weight in Equation (29) can be rewritten as:

(38) $$\begin{array}{ll} W & =(R_{N}^{-1}-\delta_{s}{}^{2}\frac{R_{N}^{-1}X(z)\cdot X^{T}(z)R_{N}^{-1}}{1+\delta_{s}{}^{2}X^{T}(z)R_{N}^{-1}X(z)})\cdot X(z)\\ & \cdot \left(X^{T}(z)(R_{N}^{-1}-\delta_{s}{}^{2}\frac{R_{N}^{-1}X(z)\cdot X^{T}(z)R_{N}^{-1}}{1+\delta_{s}{}^{2}X^{T}(z)R_{N}^{-1}X(z)})X(z)\right){}^{-1}F\\ & =R_{N}^{-1}\cdot X(z)\cdot {\left(X^{T}(z)R_{N}^{-1}X(z)\right)}^{-1}F\\ & =R_{N}^{-1}\cdot X(z)\cdot \mu^{\prime } \end{array},$$

where **μ**’∈C*^SQP×1^*. Comparing Equations (36) and (38), we find that, when the optimization problem is subjected to **W**^T^**X**(*z*) = **F** to maximize the SNR, we have **μ** = **μ**’, which means that the proposed LCMV-based multi-coil array method is equal to the maximum SNR of the borehole TEM system; therefore, the SNR, as well as the inspection performance of the downhole casings, can be effectively improved.

## 5. Field Experiments

### 5.1. Experimental Results

**Figure 3:**
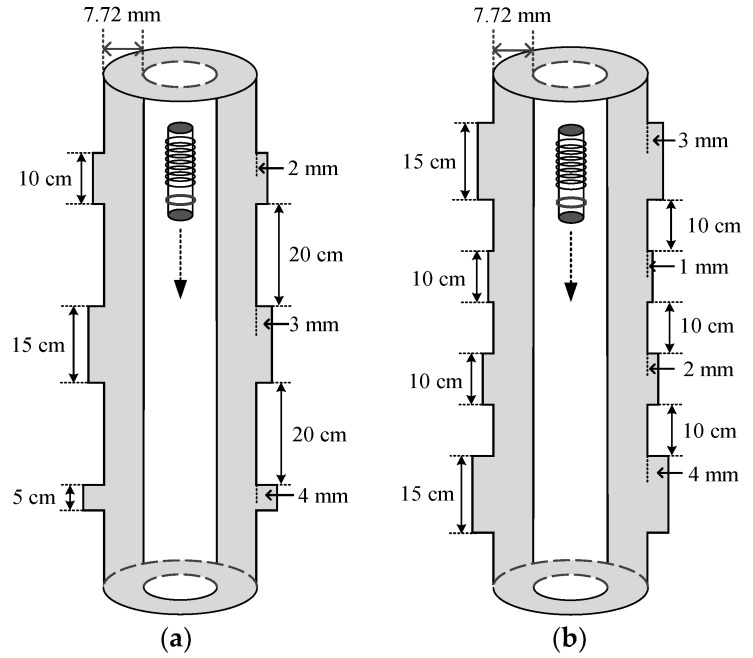
Experimental metal casing structures with different types of thickness changes: (**a**) three types of thickness changes with spacings of 20 cm (marked with casing A) and (**b**) four types of thickness changes with spacings of 10 cm (marked with casing B).

The validity of the uniform linear multi-coil array-based borehole TEM system for downhole casing inspections was confirmed using field experiments. The experiments were conducted in standardized 5^1/2^-inch (with thicknesses of 7.72 mm and outer diameters of 139.7 mm) metal casings. In our experiments, two metal casings with different structures with thickness changes, including increases in the thickness from 1 mm to 4 mm corresponding to lengths from 5 cm to 15 cm in the longitudinal direction, respectively, as shown in Figure 3, were designed and analyzed to illustrate the effectiveness of the proposed method.

The parameters of the multi-coil array sensor and the experiment are shown in Table 1, where the actual outer radius *r*_5_ can be calculated by adding the thickness change in Figure 3 to the standardized casing outer radius.

**Figure 4:**
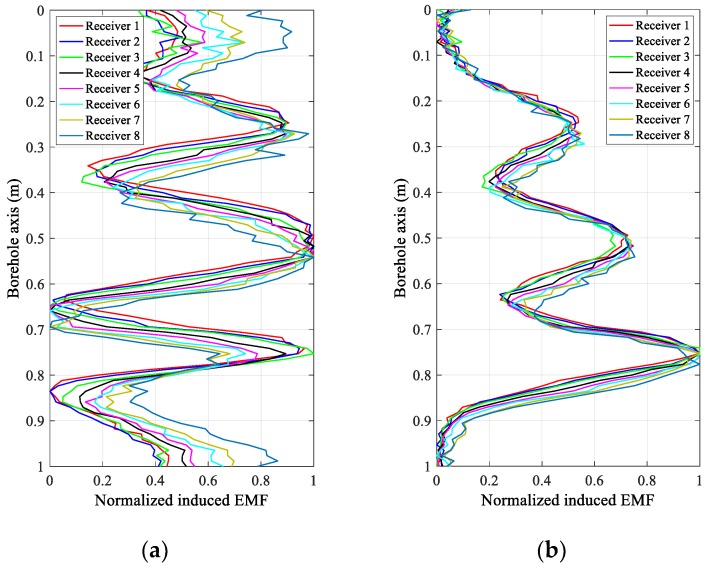
Normalized induced electromagnetic forces (EMFs) of the eight receivers of the linear multi-coil array for casing A at (**a**) 20 ms and (**b**) 40 ms.

**Figure 5:**
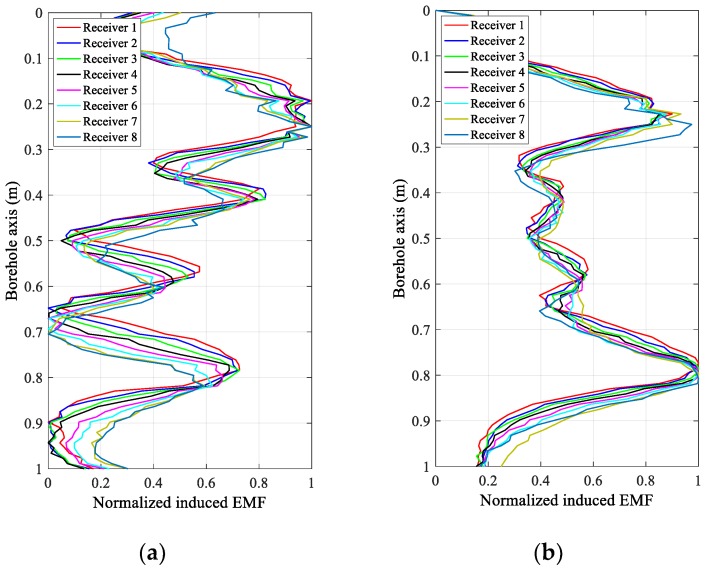
Normalized induced electromagnetic forces (EMFs) of the eight receivers of the linear multi-coil array for casing B at (**a**) 20 ms and (**b**) 40 ms.

Figure 4 and Figure 5 show the experimental results of the eight receivers of the uniform linear multi-coil array at an early time of 20 ms and a late time of 40 ms in the inspection of the experimental casing structures in Figure 3a,b, respectively. In Figure 4 and Figure 5, the received signals of each receiver are normalized to make them clearer and easier to distinguish and compare. Regardless of the decrease in the SNR with increasing TRD, we can still see that the induced EMFs of the eight receivers at the same sampling time have similar shapes, excepting a longitudinal (borehole axis) shift, which is primarily caused by the different TRDs of the different receivers, and performs just like a “phase shift” in a phased-array radar. Obviously, if the receiving data of the eight receivers are directly summed (the arithmetic mean), the resolution of the casing inspection will be substantially decreased due to the shift in the receiving data along the borehole axis with respect to the different TRDs. However, comparing the early and late time data in Figure 4 and Figure 5, we find that, even though the early time data of the TEM system have better resolution than the late time data [16], the shifting also appears more seriously, which means that the size of this type of shifting is related not only to the TRDs and the inter-element spacing but also to the eddy-current diffusion range. Therefore, it will be difficult to compensate for the shifting via an ordinary curve alignment for all the diffusion times (sampling times). Using the numerical approach in Section 3, the proposed LCMV-based array processing is only related to the TRDs and will not be affected by the sampling time and casing thickness, therefore making the compensation more effective and efficient.

### 5.2. Analysis and Discussion

In order to demonstrate the effectiveness of the proposed method, the inspection performance of a traditional borehole TEM sensor with a large number of turns in the receiving coils was also analyzed to compare to the proposed uniform linear multi-coil array sensor, where the transmitters of the two sensors were the same, and the receiver of the traditional borehole TEM sensor was approximately substituted using the arithmetic mean of the eight receivers of the linear multi-coil array, so that the number of receiving coil turns of two sensors were also the same. Note that, even though the use of a receiver with a TRD of zero (*z* = 0, collocated with the transmitter) in the linear multi-coil array can substantially improve the NDE performance, the effectiveness of the proposed method is doubtful in the case of a high SNR measurement, because the response of the collocated receiver will then be nearly the same as the desired array output. Therefore, without loss of generality, the collocated receiver is only used as a comparison to the proposed method, but is not included in the eight receivers of the uniform linear multi-coil array. Nevertheless, note that the collocated receiver, as well as the symmetric array with respect to the transmitter, will be of great importance in improving the NDE performance in a real system.

**Figure 6:**
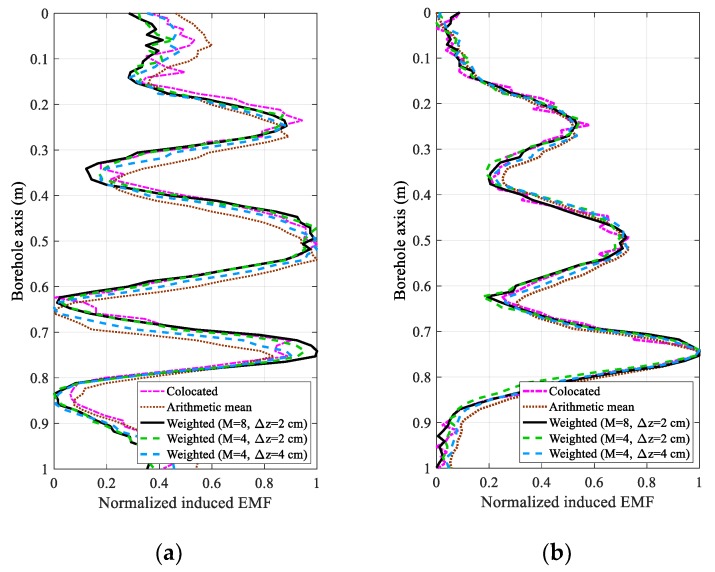
Comparison of the normalized induced electromagnetic forces (EMFs) between the traditional receiver and the linear multi-coil array for casing A at (**a**) 20 ms and (**b**) 40 ms.

**Figure 7:**
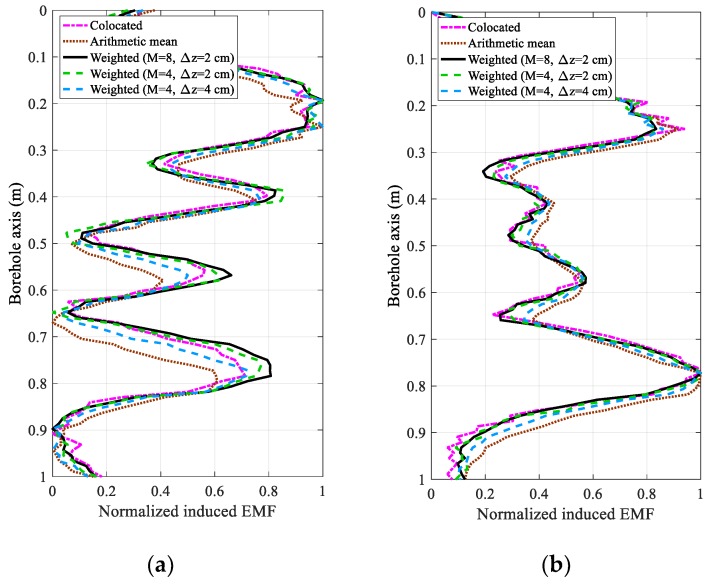
Comparison of the normalized induced electromagnetic forces (EMFs) between the traditional receiver and the linear multi-coil array for casing B at (**a**) 20 ms and (**b**) 40 ms.

Figure 6 and Figure 7 compare five cases of array output for the experimental results. The first case shows the original data of the receiver collocated with the transmitter with *z* = 0, where no shifting exists, regardless of the sampling time and motion velocity. In the second case, the induced EMFs of the eight receivers are directly summed to obtain the arithmetic mean. For comparison, we show the weighted array output using the proposed LCMV-based multi-coil array method in the last three cases, where eight receivers (Receivers 1–8) with inter-element spacings of 2 cm, four receivers (Receivers 1–4) with inter-element spacings of 2 cm, and four receivers (Receivers 2, 4, 6, and 8) with inter-element spacings of 4 cm, are employed. In this paper, we present a uniform linear multi-coil array-based borehole TEM system for the NDE of downhole casings, where the main contribution is the elimination of the influence of TRD; this paper does not focus on the interpretation algorithms. Therefore, the processed array outputs for different casing structures and different sampling times are analyzed instead of the interpreted thicknesses, because different interpretation methods using data even at different sampling times may have quite different results. Moreover, by analyzing the results produced by the proposed method with respect to the experimental casing structures, the improvement of array outputs could substantially correspond to a better NDE performance, even though the interpreted thickness changes are not illustrated.

From Figure 6 and Figure 7, we find that a serious distortion exists when the induced EMFs of the eight receivers are directly summed compared to the receiver with *z* = 0. Using the LCMV-based uniform linear multi-coil array for the borehole TEM system, the weighted array outputs in the last three cases perform more smoothly than in the first case, which reveals that a higher SNR can be achieved due to the accumulation of the coherent received signals. Notably, if the signals obtained from the first case were averaged, although there will be no phase shift and the averaging can increase the SNR substantially, repeated measurements are needed to achieve the same SNR as the proposed method, which will be quite a time-consuming procedure since the motion velocity should be slowed down compared to that of the multi-coil array-based borehole TEM system. It has also to be mentioned that since the two experimental casing structures have only one pipe per case, the amplitude of the late time data usually monotonically increases with the thickness of the metal pipe [22], and the shape of late time data will be more similar to that of the real casing thickness, as shown in Figure 3. Furthermore, comparing the experimental results for the two types of casing structures at 40 ms, the TRD will have a stronger influence on the NDE performance of Casing B than that of Casing A, which reveals that the TRD will also influence the longitudinal resolution of the NDE of downhole casings. As a result, the proposed method seems to have more effect on improving the NDE accuracy of the early time data (20 ms) and Casing A with small spacings than that of the late time data (40 ms) and Casing B with large spacings. Nevertheless, the proposed method is effective to achieve better performance for the experimental results of all cases, regardless of the casing structures and sampling times.

**Figure 8:**
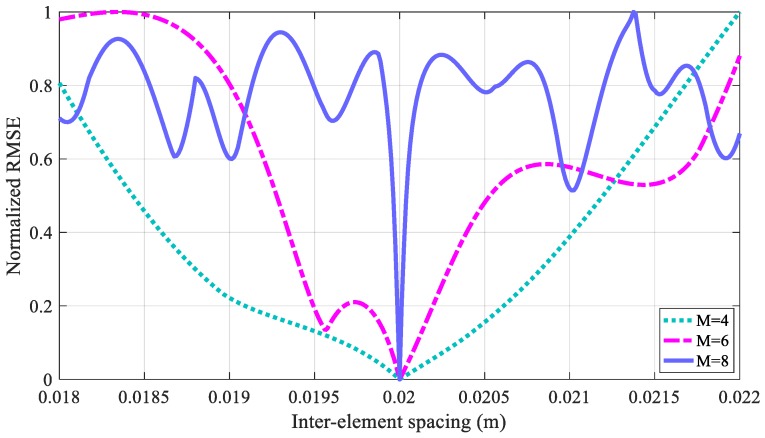
Normalized root-mean-square error (RMSE) of different inter-element spacings.

In addition, the weighted array outputs of the third and fourth cases with Δ*z* = 2 cm have nearly the same performance as the first case, where the longitudinal shifting caused by the influence of the TRD can be effectively cancelled. Furthermore, comparing the last three cases, it is obvious that the inter-element spacing Δ*z* and the number of receivers *M* influence the performance of the NDE. Because the signal strength is decreased by increasing the TRD, the SNR of each single receiver is inversely proportional to the inter-element spacing, where receivers with too large TRDs become useless. Conversely, in Section 4, it is shown that the number of receivers *M* is associated with the number of the Legendre polynomial stage *P*. Therefore, with a large value of *M*, in addition to the improved SNR due to the accumulation of the coherent signals, a large value of *P* can be achieved so that the numerical approach can approximate the borehole TEM model more closely. However, a larger number of receivers also requires a high level of accuracy in the fabrication of the uniform linear multi-coil array. Without a loss of generality, we employ the normalized root-mean-square error (RMSE) of the different inter-element spacings to further illustrate the effect of the number of receivers, where the RMSE is defined as:

(39) $$\mathrm{RMSE}=\sqrt{\frac{1}{SQP}\sum_{sqp=1}^{SQP}{\left(F_{sqp}-{\left[W^{T}X\left(z\right)\right]}_{sqp}\right)}^{2}},$$

and *F_sqp_* and [**W**^T^**X**(*z*)]*_sqp_* denote the *sqp*th element of the vectors **F** and **W**^T^**X**(*z*), respectively. The simulation results of the RMSE are shown in Figure 8.

With increasing *M*, we find that the width of the null of the RMSE curves becomes much narrower. Taking *M* = 8 as an example, even though the neighboring receivers with small inter-element spacing can be weighted to obtain a better performance, the tolerance of the proposed method for the array geometric error will also be reduced so that a high accuracy array fabrication is required, where even a 1-mm error of the inter-element spacing in the fabrication may introduce a much larger error than that of *M* = 6 or *M* = 4. Therefore, a reasonable number of receivers should consider not only the SNR and the numerical approximation, but also the array geometric error to achieve a better NDE performance. The optimization of the receiver number and the inter-element spacing of the multi-coil array need to be investigated further in future studies.

## 6. Conclusions

A uniform linear multi-coil array-based borehole TEM system was proposed to improve the NDE performance for downhole casings. We presented a Gauss–Legendre quadrature-based numerical approach for the borehole TEM signal model. It was shown that the TEM response is coupled to the TRD, which greatly influences the NDE performance. Moreover, on the basis of the proposed numerical approximation, the received signals of the uniform linear multi-coil array were weighted according to the LCMV criterion to cancel the influence of the TRD, where the optimization and performance analyses of the proposed array weighting were also investigated. Simulations and experiments for a standardized oil-well casing inspection demonstrated the effectiveness of the proposed system.

## Figures and Tables

**Table 1:** Parameters of the multi-coil array sensor and the field experiment.

Parameter	Symbol	Value
Radius of the multi-coil array sensor	*r*_1_	12 mm
Number of receiving coils	*M*	8
Inter-element spacing	Δ*z*	20 mm
Transmitting–receiving distances	z_1_–z*_M_*	20–160 mm
Number of transmitting coil turns	*N*_T_	19
Number of receiving coil turns	*N*_R_	62
Tool housing inner radius	*r*_2_	18.5 mm
Tool housing outer radius	*r*_3_	21.5 mm
Standardized casing inner radius	*r*_4_	62.13 mm
Standardized casing outer radius	*r*_5_	69.85 mm
Cement ring outer radius	*r*_6_	88.9 mm
